# Rapamycin Blocks Production of KSHV/HHV8: Insights into the Anti-Tumor Activity of an Immunosuppressant Drug

**DOI:** 10.1371/journal.pone.0014535

**Published:** 2011-01-14

**Authors:** Lisa A. Nichols, Laura A. Adang, Dean H. Kedes

**Affiliations:** 1 Myles H. Thaler Center for AIDS and Human Retrovirus Research, University of Virginia, Charlottesville, Virginia, United States of America; 2 Department of Microbiology, University of Virginia, Charlottesville, Virginia, United States of America; 3 Department of Medicine, University of Virginia, Charlottesville, Virginia, United States of America; Duke University, United States of America

## Abstract

**Background:**

Infection with Kaposi's sarcoma-associated herpesvirus (KSHV/HHV8) often results in the development of fatal tumors in immunocompromised patients. Studies of renal transplant recipients show that use of the immunosuppressant drug rapamycin, an mTOR inhibitor, both prevents and can induce the regression of Kaposi's sarcoma (KS), an opportunistic tumor that arises within a subset of this infected population. In light of rapamycin's marked anti-KS activity, we tested whether the drug might directly inhibit the KSHV life cycle. We focused on the molecular switch that triggers this predominantly latent virus to enter the lytic (productive) replication phase, since earlier work links this transition to viral persistence and tumorigenesis.

**Methods and Findings:**

In latently infected human B cell lines, we found that rapamycin inhibited entry of the virus into the lytic replication cycle, marked by a loss of expression of the lytic switch protein, replication and transcription activator (RTA). To test for viral-specific effects of rapamycin, we focused our studies on a B cell line with resistance to rapamycin-mediated growth inhibition. Using this line, we found that the drug had minimal effect on cell cycle profiles, cellular proliferation, or the expression of other cellular or latent viral proteins, indicating that the RTA suppression was not a result of global cellular dysregulation. Finally, treatment with rapamycin blocked the production of progeny virions.

**Conclusions:**

These results indicate that mTOR plays a role in the regulation of RTA expression and, therefore, KSHV production, providing a potential molecular explanation for the marked clinical success of rapamycin in the treatment and prevention of post-transplant Kaposi's sarcoma. The striking inhibition of rapamycin on KSHV lytic replication, thus, helps explain the apparent paradox of an immunosuppressant drug suppressing the pathogenesis of an opportunistic viral infection.

## Introduction

The tumorigenic virus Kaposi's sarcoma-associated herpesvirus (KSHV, human herpesvirus 8 or HHV8) is the causative agent of primary effusion lymphoma (PEL), multicentric Castleman's disease (MCD), and, most commonly, Kaposi's sarcoma (KS) [1], [2]. KSHV, as with all herpesviruses, has both a latent phase in which the virus expresses few proteins, as well as a lytic phase during which virion production occurs. While the latent form of viral infection is predominant both in KS lesions as well as within PEL cells, maintenance of KSHV infection and subsequent tumorigenesis in the setting of immunosuppression are dependent on viral lytic replication and the subsequent infection of naïve target cells by newly released virions [3], [4]. Replication and transcription activator (RTA), encoded by KSHV open reading frame (ORF) 50, initiates the lytic protein cascade [5]–[7]. Moreover, expression of RTA is necessary and sufficient for commencement of lytic replication [6]. In the laboratory setting, the addition of specific chemical agents to latently infected cells induces lytic reactivation. Valproic acid (VPA), for example, activates KSHV likely through its role as a histone deacetylase (HDAC) inhibitor [8]. KSHV also reactivates in the presence of phorbol esters, such as 2-O-tetradecanoyl-phorbol-13-acetate (TPA), that upregulate the Raf/MEK/ERK pathway [9] and cobalt chloride, a hypoxia mimetic, that elevates levels of hypoxia inducible factor-1 alpha (HIF-1α) [10], [11]. While these three induction pathways ultimately result in increased RTA expression, it is unclear whether these signaling pathways are independent or, instead, share regulatory control points upstream of RTA.

Recent reports have linked the immunosuppressant rapamycin (sirolimus) to the regression of KS in renal transplant patients. Since KSHV-induced diseases arise and progress primarily in immunocompromised populations, the inhibition of PEL-like tumors in an animal model using this treatment appears counter-intuitive [12]–[19]. Rapamycin acts via the inhibition of the mammalian target of rapamycin (mTOR). mTOR is a highly conserved kinase and a central component in signaling cascades that modulate a wide range of metabolic processes. It is particularly critical in promoting protein synthesis and cell cycle progression (as reviewed in [20]). Pharmacological inhibition of mTOR using rapamycin, therefore, can have a wide range of effects, and significantly, may have a pronounced anti-neoplastic effect on cells or tumors whose growth is dependent on high levels of mTOR activity. Thus, it is noteworthy that other groups have found that the mTOR pathway is highly active in KSHV-infected cells and contributes to cell survival, growth and production of angiogenic factors [15], [21]. However, in light of the sensitive balance between immune health and gammaherpesvirus induced tumors, even the anti-proliferative effects of rapamycin seem inadequate to fully explain the suppression and even regression of KSHV-induced pathogenesis. Of particular interest is a clinical study by Barozzi et al. wherein patients with post-transplant KS treated with rapamycin show a drop in virus copy number that, remarkably, precedes both the enhancement of KSHV-specific immune responses and tumor regression [22]. This suggests that either cells with lytic viral replication are particularly susceptible to rapamycin treatment or, instead, there is a direct suppression of virion production from KSHV^+^ cells. We asked whether the drug might play a more direct role in suppressing KSHV itself. We investigated whether the lytic phase of the KSHV lifecycle is especially sensitive to the inhibitory actions of rapamycin. We hypothesized that rapamycin may block the essential transition of KSHV from latency to lytic replication since low-level entrance into this second phase of the viral lifecycle is essential for survival of the virus in vivo and in vitro [3], [4].

To test this hypothesis, we employed primary effusion lymphoma cell lines, a tissue culture based system, for viral reactivation. Consistent with its anti-neoplastic role, rapamycin can induce cell cycle arrest in a number of KSHV^+^ lymphoma cell lines [14]. Therefore, to test the role of mTOR signaling blockade specifically on viral protein expression and virion production, we utilized a PEL line that is refractory to rapamycin-dependent growth perturbations. We found that mTOR played an essential role in RTA expression, as rapamycin was sufficient to block reactivation in the majority of cells irrespective of the route of lytic induction.

## Methods

### Cell culture

BCBL-1 cells [23] were grown in RPMI 1640 media (Gibco) supplemented with 10% fetal bovine serum (FBS), 10 mM HEPES (pH 7.5), 100 U/ml penicillin, 100 µg/ml streptomycin, 2 mM L-glutamine, 0.05 mM β-mercaptoethanol, and 0.02% w/v sodium bicarbonate. HeLa (American Type Culture Collection) cells were grown in D-MEM (Gibco) media supplemented with 5% FBS and 2 mM L-glutamine.

### Drug treatments

Virion production was induced with cobalt chloride (CoCl_2_) (100 µM), O-tetradecanoylphorbol-13-acetate (TPA) (20 ng/mL), or valproic acid (VPA) (300 or 600 µM). Cells were pre-treated with indicated dose of Rapamycin (EMB Bioscience) or DMSO vehicle control 2 hours prior to lytic induction.

### Infection with KSHV

Virus was collected from induced BCBL-1 cells on the fifth day after induction by centrifugation at 13,000x*g* for three hours as previously described [24]. The viral pellet was resuspended in one-two hundredth the original volume with PBS, and the concentrated virus (stored at 4°C for up to 1 week without loss in titer) was used to infect target HeLa cells in the presence of 8 µg/ml polybrene (Sigma Aldrich). For cell-associated infection, BCBL-1 were treated with rapamycin, then on day 4 were plated with HeLa cells at a ratio of 6∶1 (BCBL-1: HeLa) overnight in the presence of 8 µg/ml polybrene. HeLas were washed twice to remove BCBL-1 cells, then trypsinized, fixed and permeabilized with ice-cold MeOH/Acetone, rehydrated in ICSB and stained for intracellular LANA. Rat anti-LANA antibody (Advanced Biotechnologies) directly conjugated to AlexaFluor488 (Molecular Probes) was used at 1∶1000. Just prior to analysis cells were stained with DRAQ5 at 50 µM to allow visualization of nuclei (BioStatus Ltd, United Kingdom). Samples were run on the ImageStream100 (Amnis Corp., Seattle, WA) and analyzed using IDEAS software 3.0 (Amnis) to count number of LANA dots within nuclei of infected HeLa cells.

### DNA content analysis (cell cycle)

BCBL-1 cells were harvested two days following drug treatments, washed once with cold 1x PBS, and resuspended in cold 70% ethanol, followed by a two-hour (minimum) incubation. Cells were then pelleted and resuspended in PBS, incubated for 1 minute, and re-pelleted before resuspending in PI staining solution (0.1% v/v Triton X-100 in Tris buffered saline, 20 U/mL DNase-free RNase A, and 5 mg/mL propidium iodide) and incubating for 30 minutes at 20°C or 15 minutes at 37°C. DNA content was measured on a FACS Calibur Flow Cytometer (BD) and analyzed using the Modfit LT software (Verity Software House) [25].

### Flow cytometry

BCBL-1 were harvested for analysis by flow cytometry at the indicated times. Cells were resuspended in PBS and stained with Live/Dead fixable Violet or Aqua exclusion dye (Molecular Probes, Invitrogen) per manufacturer instructions to identify viable cells. Cells were then fixed with 2% paraformaldehyde for 15 min., resuspended in cold Intracellular Staining Buffer (ICSB; 0.2% Sodium Azide and 1% BSA in PBS), fixed with 2% paraformaldehyde for 15 minutes and permeabilized in 1X Perm/Wash (BD Biosciences). Mouse monoclonal RTA antibody (a kind gift from K. Ueda) was diluted 1∶2000 in Perm/Wash. Anti-ORF59 and K8.1 monoclonal antibodies (Advanced Biotechnologies) were conjugated to Pacific Blue or Alexa Fluor647 fluorchromes (Invitrogen) and titered before use at 1∶500 and 1∶5000 dilutions, respectively. Polyclonal anti-mouse secondary antibody conjugated to APC or to Cy3 (Jackson ImmunoResearch) was diluted 1∶500 in Perm/Wash. Flow cytometry was performed with a FACS Calibur Flow Cytometer (Becton Dickinson) or CyAn ADP LX (Beckman Coulter) and analyzed with FlowJo software (Tree Star). Prior to analysis, all samples were gated using forward and side scatter to exclude cellular debris.

### Immunoblot

Total cellular proteins were harvested as previously described [26]. Following a wash in 1x PBS, the cell pellets (approximately 3×10^7^ cells) were washed with cytoplasmic lysis buffer (10 mM HEPES pH 7.9, 1.5 mM MgCl_2_, 10 mM KCl, 0.5 mM dithiothreitol [DTT], 0.2 mM phenylmethylsulfonyl fluoride [PMSF], and 1x protease inhibitor cocktail [Roche] with protease inhibitors). The pellets were resuspended in cytoplasmic lysis buffer, placed on ice for 10 minutes, vortexed for 30 seconds, and pelleted. This swell-vortex-pellet step was repeated prior to the resuspension of the nuclear pellet in the nuclear lysis buffer (10 mM HEPES pH 7.9, 25% glycerol, 42 mM NaCl, 1.5 mM MgCl_2_, 0.2 mM EDTA, 0.5 mM DTT, 0.2 mM PMSF, and 1x protease inhibitor cocktail). Nuclei were resuspended in roughly equal volume of nuclear lysis buffer. Following a 15-minute incubation on ice, DNA was pelleted and the nuclear extracts were recovered. Approximately 1–5 mg of protein as determined by modified Bradford Assay (Bio Rad) was loaded into each lane of a 10% Bis-Tris gel (Invitrogen). Rabbit anti-mTOR (Sigma; 1∶1000), rabbit anti-S6K (Santa Cruz Biotechnologies, Inc; 1∶1000), rabbit anti-phospho-S6K (Sigma), mouse anti-a-tubulin (Sigma; 1∶2000); goat anti-RCC1 (Santa Cruz Biotechnologies, Inc; 1∶2000), mouse anti-HIF1-a (BD Biosciences; 1∶400), mouse anti-vIRF3 (a kind gift from Y. Chang; 1∶1000), and rabbit anti-RTA (a kind gift from D. Lukac; 1∶2000) were used to probe the nitrocellulose membrane (Bio Rad). All antibodies were diluted in 5% milk in TBS-Tween (0.5%) and secondary HRP-conjugated antibodies were used at 1∶10,000. Non-enzymatic immunoblots were stained with anti-mouse or anti-rabbit IgG antibodies conjugated to Infrared Dye 680 or 800 and images scanned and analyzed using Odyssey Infrared Imaging System and 3.0 Software (Licor Biosciences).

### Real-time PCR

Total RNA was isolated from BCBL-1 cells using Qiagen RNeasy RNA Isolation Kit (Qiagen Inc., Valencia, CA). Briefly, cells were pelleted, supernatant removed and pellet resuspended in RLT lysis buffer. Lysates were homogenized by loading onto Qiashredder columns (Qiagen, Inc.), spinning at 13,000K x 2 min. Homogenized lysates were stored at −80°C until further purification. Total RNA was treated with on-column DNase (Qiagen, Inc) and again after elution from RNA columns using Ambion DNA-free kit (Life Technologies, Carlsbad, CA). cDNA was generated from 1 ug of RNA using Omniscript reverse transcriptase (Qiagen) and oligo-dT primers to generate template for GAPDH quantification and an ORF50-specific reverse primer (5′ GGAGAGGGACTACTGTTGG 3′) to generate template for ORF50 mRNA quantification. Real-time PCR using forward and reverse DNA primers (IDT) and labeled FAM-BHQ1 probes (Biosearch Technologies) was done in triplicate on diluted cDNA using Taqman Universal PCR Mix (Applied Biosystems) and analyzed by UVA DNA Sciences using an ABI Prism® 7900 HT Detection System.

ORF50 primer/probe set:

Fwd – TTGTCGCAGAGAACACCGG

Rev – GCAAGGGTGACATGACGTCA

Probe – CAAGCTTCCCGTTCTCAGCC

GAPDH primer/probe set:

Fwd – GATTCCACCCATGGCAAATT

Rev – GAAGATGGTGATGGGATTTCCA

Probe – CAAGCTTCCCGTTCTCAGCC

All ORF50 transcripts were normalized to GAPDH for analysis.

### Statistical analyses

Population means were assessed for statistical significance using an unpaired t test to calculate two-tailed p values. All analyses were done using the built-in statistical algorithms in GraphPad Prism 4.0 software. The results from these analyses are stated in the legends or indicated within the figure. P values less than 0.05 were considered statistically significant.

## Results

### Rapamycin blocks S6K phosphorylation in BCBL-1 cells without substantial changes in cell proliferation or cell cycle

**Figure 1:**
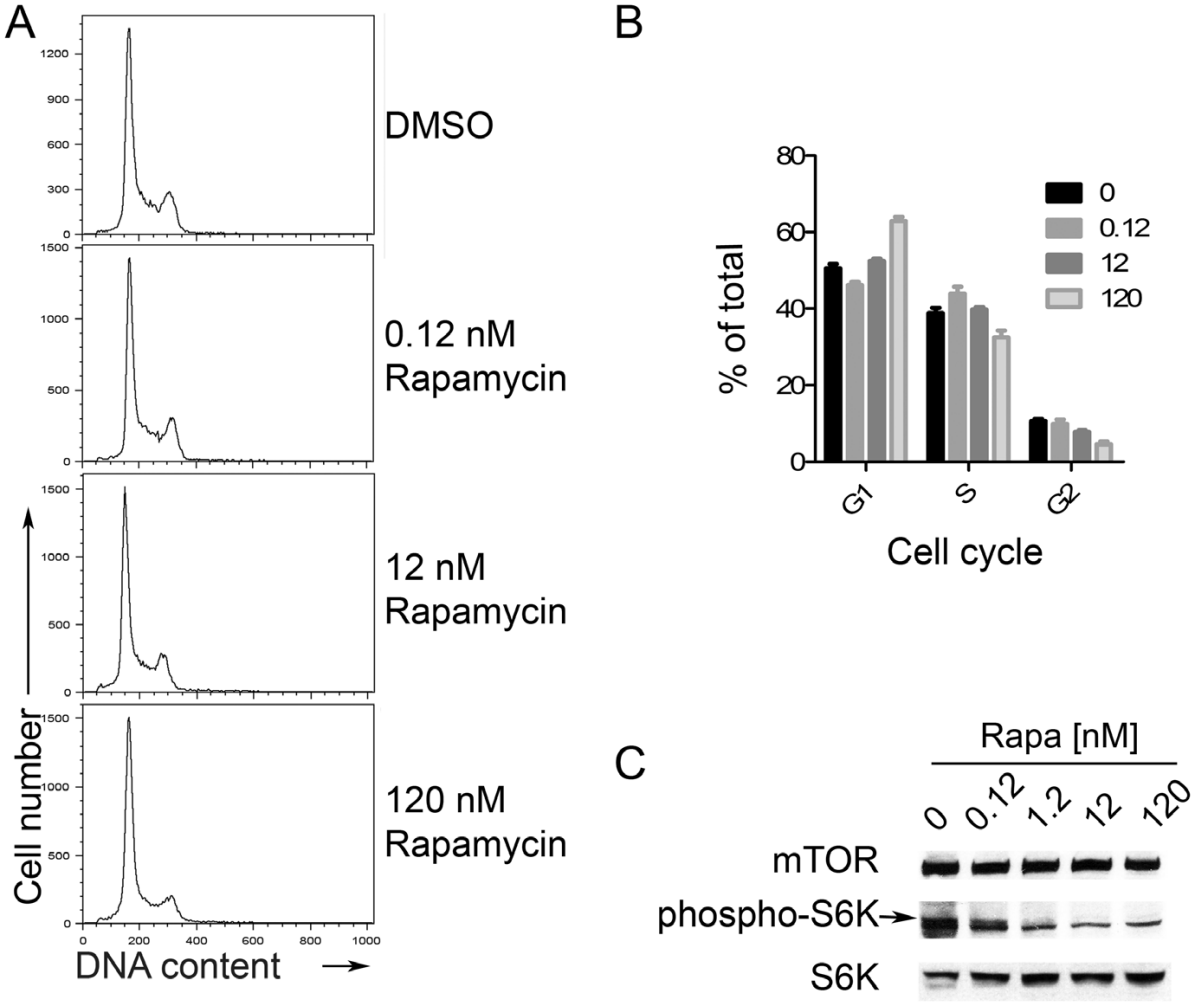
Rapamycin blocks S6K phosphorylation in BCBL-1 cells, but does not block cell cycle progression. (A) DNA content analysis of BCBL-1 cells treated with rapamycin (0.12 nM, 12 nM, 120 nM) or DMSO vehicle control was determined by PI staining (representative experiment of n≥3). (B) Graph of percentage of cells in either the G1, S, G2 subset as determined using the Modfit LT analysis software. (C) BCBL-1 cells treated with rapamycin at indicated doses. At 48 hours post-treatment, whole cell extracts were immunoblotted for mTOR, S6K phosphorylated at Thr^389^, and S6K.

mTOR can play an important role in regulating the cell cycle and rapamycin, an inhibitor of mTOR signaling, appears to control the growth of several KSHV-associated tumors [14]. However, not all types of KSHV^+^ tumors are comparably susceptible to rapamycin-mediated growth inhibition and, importantly, the viral lytic (productive) cascade is a critical component of pathogenesis for KSHV^+^ tumors [3], [4]. Thus, we sought to determine whether in KSHV^+^ cells, treatment with rapamycin might also directly affect the viral life cycle. To separate the effects of cell cycle perturbation from changes in viral gene expression, we conducted experiments using a cell line that is relatively resistant to profound growth arrest with rapamycin treatment[14]. We verified the rapamycin resistance of this KSHV^+^EBV^-^ primary effusion lymphoma cell line, BCBL-1 [23], to growth arrest by treating with the drug and assessing cell cycle profiles. Following 48 hours of treatment of BCBL-1 with a range of rapamycin doses, we harvested and then stained the cells with the DNA dye propidium iodide to measure cellular DNA content using flow cytometry. We found that increasing amounts of rapamycin minimally affected the cell cycle profile, though at the highest dose, 120 nM, some inhibition was evident (Figure 1A and B). These results contrasted, not surprisingly, with the effects of the protein synthesis inhibitor, cycloheximide, which caused dose-dependent decreases in the proportion of the population in S phase and concurrent accumulation in G1 and G2/M (not shown) [27], [28]. To verify the inhibition of mTOR function in these cells despite the lack of cell cycle arrest, we assessed inhibition of mTOR function in drug-treated cells by immunoblotting for phosphorylation of its downstream target, S6 kinase. As with other cell types[29]–[31], treatment of BCBL-1 cells with rapamycin for two days resulted in a specific loss of phosphorylated p70 S6 kinase (S6K) at position Thr^389^ (Fig. 1C).

### Spontaneous and induced RTA expression is decreased in rapamycin-treated BCBL-1 cells

**Figure 2:**
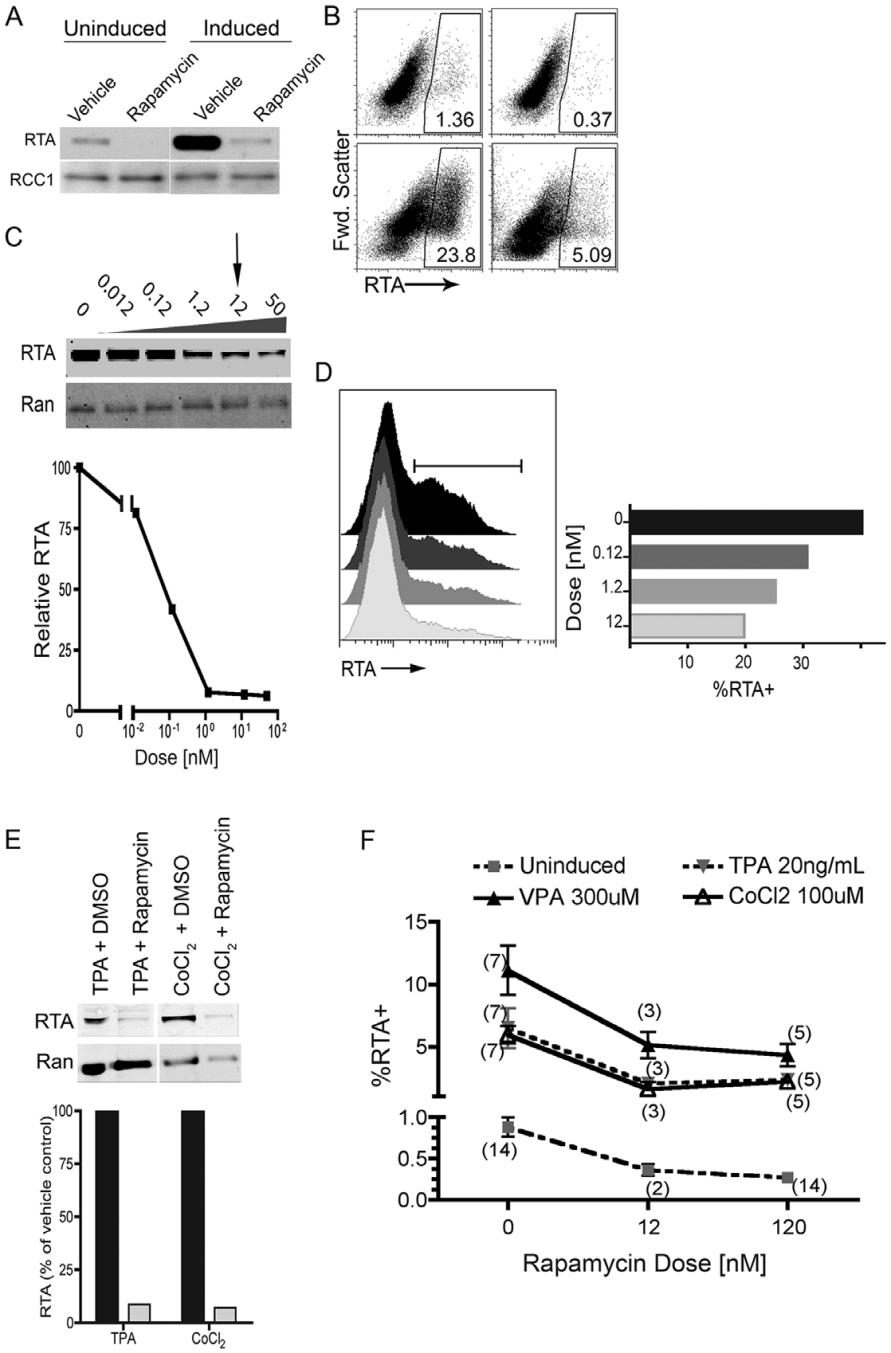
Rapamycin inhibits spontaneous and induced RTA expression in a dose-dependent manner regardless of induction pathway. BCBL-1 were treated with 120 nM rapamycin or vehicle for 2 h, and then without (uninduced) or with 0.6 mM VPA (induced). (A) 48 h post-treatment, nuclear extracts were analyzed by immunoblot for RTA expression using the nuclear protein, RCC1, as a loading control. Representative experiment, n = 3. (B) 48 h post-rapamycin, uninduced BCBL-1 cells (top panels) or VPA-induced (bottom panels) were harvested, treated with a dead cell stain, then fixed, stained for intracellular RTA, and analyzed by flow cytometry. Representative plots (n = 7) show RTA expression in live-gated BCBL-1 cells treated with vehicle (left panels) or rapamycin (right panels). (C) Nuclear extracts were analyzed for RTA expression using non-enzymatic infrared detection probes to quantify relative protein levels. Ran (ras-related nuclear protein) was used as loading control. Graph (C, bottom panel) shows immunoblot RTA levels normalized to Ran as a percentage of RTA levels in the vehicle (DMSO) treated control. Representative experiment (n = 2). (D) Induced BCBL-1 cells treated for 48 h with rapamycin at indicated doses were fixed, stained for intracellular RTA and analyzed by flow cytometry. Graph, right, shows percent of RTA^+^ cells in population indicated by histogram gate. (E) BCBL1 48 h post-treatment cells were harvested and nuclear extracts immunoblotted for RTA and normalized to Ran. Graph shows quantification of bands using non-enzymatic infrared detection probes. Representative experiment (n = 2). (E) BCBL-1 treated with indicated doses of rapamycin and left uninduced (square, dashed line), or induced with either VPA (triangle, solid line), TPA (triangle, dashed line), or CoCl_2_ (open triangle, solid line) were cultured for 48 hrs in presence of rapamycin, then stained with intracellular RTA. Graph shows percentage of RTA^+^ cells in live cell population. Mean ± s.e.m.; n for each condition shown in parentheses.

Clinical observations suggest that active viral production of KSHV is associated with pathogenesis and that rapamycin inhibits the formation of Kaposi's sarcoma [3], [13], [32]–[36]. Therefore, we next investigated the potential role of mTOR (and its blockade by rapamycin) in lytic replication of the virus. Using RT-PCR, Sin and colleagues found that rapamycin has no discernable effect on baseline levels of viral transcripts among latently infected PEL lines [14]. As a result, we hypothesized that mTOR inhibition may modulate lytic KSHV replication through post-transcriptional effects; therefore, we examined viral protein levels in the presence and absence of mTOR inhibition. As RTA is both necessary and sufficient for the initiation of the lytic cascade, we focused our attention on determining the effects of rapamycin on levels of this critical latent-to-lytic switch protein [5], [6]. After treating BCBL-1 cells with rapamycin for two days, we employed immunoblots to assess relative levels of RTA within nuclear protein extracts, using the nuclear protein RCC1 (regulator of chromosome condensation 1) as a loading control (Figure 2A). We found a low level of spontaneous RTA expression in the vehicle treated BCBL-1 population; however, with rapamycin treatment, RTA became undetectable. We verified this finding using intracellular antibody staining and flow cytometric analysis, observing a greater than 50% reduction in the number of BCBL-1 cells spontaneously expressing RTA at detectable levels (Fig. 2B, top panels). We next tested the ability of rapamycin treatment to suppress RTA levels in the presence of a potent lytic cycle inducing agent, VPA. As we expected, addition of VPA to BCBL-1, in the absence of mTOR inhibition, led to a robust increase in RTA levels by 48 hrs. In stark contrast, pre-treatment with rapamycin almost completely blocked this induction (Fig. 2A). Again, we confirmed this drop in RTA expression by flow cytometric analysis of live cells stained for RTA expression (Fig. 2B, bottom).

### Rapamycin inhibition of RTA expression is dose-dependent and independent of the induction reagent

To assess more precisely the degree of RTA inhibition by rapamycin, we treated BCBL-1 cells with increasing concentrations of the drug and quantified RTA levels using non-enzymatic infrared detection and analysis of immunoblot bands (Fig. 2C). Rapamycin, at doses as low as 1.2 nM, reduced average RTA levels by more than 90%. Since 12 nM rapamycin more closely approximates the target serum trough levels maintained in patients during treatment [15], [18], [37], we used this dose for most of our experiments. Again, we verified the immunoblot results at the single cell level using flow cytometric analysis and found the proportion of BCBL-1 cells expressing RTA decreased with increasing concentrations of rapamycin (Fig. 2D). Although only limited information exists for the mechanisms involved in chemical reactivation of KSHV, lytic cycle induction by VPA depends on its functions as an HDAC inhibitor [8]. To determine whether the suppression of lytic gene expression by rapamycin was limited to this specific type of reactivation stimulus, we tested BCBL-1 treated with either TPA, which activates the ERK/MAPK pathway, or CoCl_2_, which acts as a hypoxia mimetic (activating RTA via HIF1α) to induce RTA expression [10], [11]. Briefly, we treated BCBL-1 with rapamycin and then, following incubation with either TPA or CoCl_2_, used immunoblots (Fig 2E) to measure levels of RTA expression. Regardless of the lytic inducing drug or its dose, we detected a dramatic loss of RTA expression in rapamycin treated cells. We verified these immunoblot results using flow cytometry to detect intracellular RTA staining across multiple experiments (Fig. 2F). Of note, we also consistently observed that in the absence of any inducing agent, rapamycin resulted in the loss of baseline (spontaneous) RTA expression (Fig. 2A,B and E). Together, these results suggested that mTOR might play an essential role in both induced and, perhaps more clinically significant, spontaneous mechanisms of lytic reactivation.

**Figure 3:**
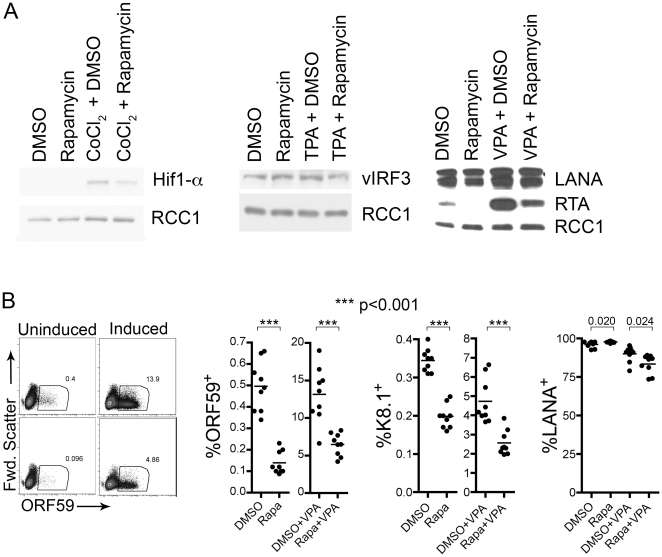
Rapamycin treatment significantly decreases induction of lytic KSHV proteins. (A) BCBL-1 cells were pre-treated for 2 hours with rapamycin (12 nM), then induced, in the presence of rapamycin with either CoCl_2_ or TPA. (A) BCBL-1 cells were pre-treated for 2 hours with rapamycin 120 nM or DMSO vehicle control two hours prior to induction with CoCl_2_ (200 mM), TPA (20 ng/mL) or VPA (600 µM). Nuclear extracts were analyzed by immunoblot for HIF1α expression 24 hours (left), or vIRF3 expression (middle) or LANA expression (right) 48 hours post-treatment. (B) Uninduced or VPA-induced BCBL-1 cells were harvested 48 h post-rapamycin treatment (12 nM), then fixed and stained with antibodies against ORF59, K8.1 and LANA. Leftmost set of dot plots show ORF59 staining on representative samples from uninduced (left panels) and induced (right panels) BCBL-1 cells treated with either vehicle (top panels) or rapamycin (bottom panels). Similar data was collected for both K8.1 and LANA expression. Graphs depict results from multiple replicates of these experiments and indicate the percent of live-gated cells positive for indicated protein stain; horizontal lines depict the mean of replicates (individual circles). Numbers above graph depict p values of comparisons.

We next investigated the potential effect of rapamycin (and loss of mTOR activity) on the expression of other proteins to assess its relative specificity for RTA expression. We asked whether inhibition of RTA expression simply reflected a global shutdown of all protein synthesis within the treated cells. To address the possibility that some proteins might have long half-lives, potentially confounding our analyses of rapamycin's effects, we took advantage of previously established data demonstrating that CoCl_2_ results in the de novo transcription and subsequent protein expression of both RTA and HIF1α [10], [11]. In contrast to the profound suppression of CoCl_2_-induced RTA expression with the lowest doses of rapamycin (Figure 3A), CoCl_2_-induction of HIF1α was only minimally affected even with the highest doses (120 nM) of rapamycin (Figure 3A, left panel; we also obtained similar results at 48 hours). We next assessed the two latent proteins, viral interferon regulatory factor-3 (vIRF-3) and the latency-associated nuclear antigen (LANA). Immunoblots revealed that rapamycin treatment (with or without induction) had no appreciable effect on these two proteins (Figure 3A, middle and right). We predicted that rapamycin-suppression of RTA would result in commensurate changes in downstream lytic genes. Flow cytometry, in fact, revealed that rapamycin treatment led to reductions ranging from 43 to 72 percent in the levels of both the delayed-early protein ORF59 (processivity factor) and the late glycoprotein K8.1. This effect was evident in both uninduced and induced cultures (see Figure 3B dot blot for ORF59 and scatter graph representations for ORFs 59 and 8.1). In contrast, rapamycin had little effect on the percentage of cells positive for LANA (Figure 3B, rightmost panel). (Although the differences in LANA expression with and without rapamycin reached statistical significance, the variation was under 8%.) Thus, while mTOR can regulate multiple cellular response pathways, rapamycin appeared to have a disproportionate impact on RTA and its downstream lytic genes compared to cellular or latent viral proteins.

### Rapamycin mediates its inhibition of RTA expression via both transcriptional and post-transcriptional mechanisms

**Figure 4:**
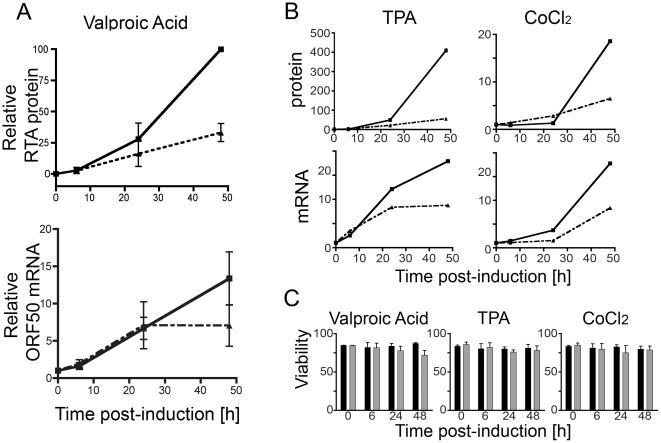
RTA regulation by rapamycin is mediated at both the mRNA and protein level. Messenger ORF50 RNA and RTA protein levels were assessed in BCBL-1 pre-treated with rapamycin for 2 hours, and then induced to lytic reactivation using VPA, TPA, or CoCl_2_. Samples were collected at 0, 6, 24, and 48 hours post-treatment. (A) Top panel, nuclear extracts were used to determine RTA protein levels at each time point using non-enzymatic, immunoblot quantification and normalization to Ran. Graphs show mean ± s.e.m. of triplicate experiments for DMSO (solid line) and rapamycin-treated (dashed line) samples. Individual experiments were normalized to the maximal protein expression levels in DMSO sample at 48 h post-induction. Bottom panel, total mRNA was also collected for quantitative RT-PCR analysis of ORF50 mRNA from parallel whole lysate samples. Graph shows pooled data from 3 experiments with DMSO (solid line) and rapamycin-treated (dashed line) cultures shown. Means ± s.e.m. (B) BCBL-1 cells treated with rapamycin (dashed lines) or DMSO (solid lines) were induced with either TPA (left panels) or CoCl_2_ (right panels). RTA protein levels (top) and mRNA levels (bottom) are shown from representative experiments. (C) For each timepoint and induction, viability was determined for both DMSO (black bar) and rapamycin-treated (gray bar) samples by staining an aliquot from each culture with a live/dead exclusion dye and assessing for dye uptake (cell death) by flow cytometry. Graphs show % viable, mean ± s.e.m. of triplicates.

Although the metabolic effects of mTOR inhibition are protean, much of the research in this area has focused on the resulting down-regulation of translation. Therefore, we asked if the relative decrease in RTA protein levels in rapamycin treated BCBL-1 cells occurred in the absence of decreases in ORF50 (RTA) mRNA levels. We measured both ORF50 mRNA and RTA protein levels in cultures pre-treated with rapamycin for 2 hours and then incubated with VPA to induce lytic reactivation of KSHV (Fig. 4A). We collected samples at 0, 6, 24, and 48 hours post-VPA addition and then determined RTA protein levels by quantitative immunoblotting (Fig 4A, top) and, in parallel samples, ORF50 mRNA levels by quantitative RT-PCR (Fig 4A, bottom). While the magnitude of RTA expression was variable between experiments compared to vehicle-treated samples, rapamycin consistently suppressed RTA levels at the 24-hour time point while the ORF50 mRNA levels remained essentially unchanged. This suggested, as we had expected for mTOR inhibition, that a post-transcriptional block at least partially mediated the decrease in RTA protein. By 48 hours, the rapamycin suppression of RTA levels was even more pronounced but we also observed an approximately 2-fold decrease in ORF50 mRNA levels within the drug-treated populations.

Although for VPA-induced cells the drop in protein appeared to precede change in mRNA, we noted that for TPA- and CoCl_2_-induced cells, there was a modest reduction (mean 1.6 and 2.4 fold, respectively) in mRNA at 24 h (Fig. 4B). Therefore, we were unable to exclude transcriptional regulation of RTA expression during the activation of these pathways in the presence of rapamycin. Regardless of induction method, we noted an approximate 2-fold decrease in ORF50 mRNA and a 2- to 10-fold drop in RTA protein levels by 48 hours (Fig. 4).

Since previous work has shown that rapamycin can directly arrest growth of a number of cancer lines, including a subset of PEL lines, we ascertained that the observed decreases in mRNA and protein did not reflect overall cell viability. Cell viability in DMSO versus rapamycin treated cultures was similar for all inducing conditions and time points (Fig. 4C). Together, the data suggested that rapamycin significantly inhibited RTA expression relative to other cellular and viral proteins and that a combination of transcriptional and post-transcriptional effects rather than a loss of cell viability or global suppression mediated this inhibition.

### Commensurate reduction in release and transmission of infectious virions

**Figure 5:**
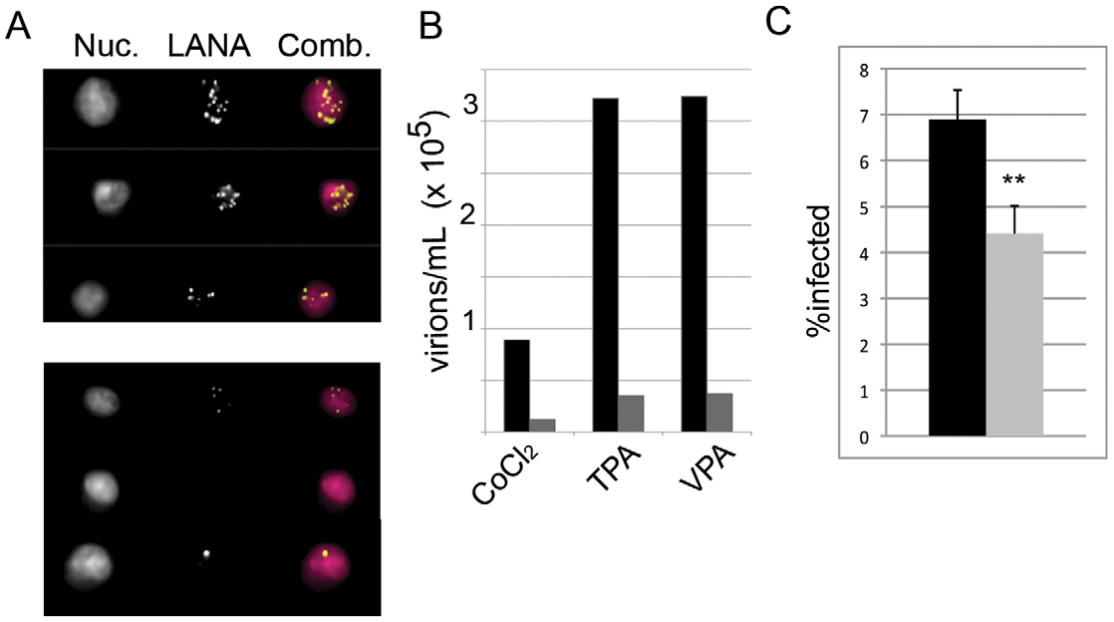
Virion production is significantly decreased by rapamycin treatment. BCBL-1 +/− rapamycin 12 nM were induced with CoCl_2_, TPA, or VPA. Five days post-induction, virus was concentrated from supernatants. HeLa cells were then infected with concentrated virus in the presence of polybrene. Viral titers were determined by staining HeLa cells for punctate intranuclear LANA dots and analyzing via multispectral imaging flow cytometry. (A) Images show representative nuclear staining with DRAQ5 (Nuc.), LANA antibody stain, and the overlay (Comb.). Upper panel shows three representative HeLa cells infected with inoculum from untreated VPA-induced BCBL-1. Lower panel shows representative HeLas infected with inoculum from rapamycin-treated VPA-induced cells. (B) Graph of viral titers untreated (black bars) or treated with rapamycin (gray bars) for each indicated inducting agent. Representative experiment, n = 2. (C) Inhibition of cell-to-cell virus transmission from spontaneously lytic BCBL-1 was assessed by culture of BCBL-1 in the presence of 12 nM rapamycin for 2 days. Cells were then harvested, placed in fresh media without rapamycin and co-cultured with HeLa cells for 24 h. BCBL-1 cells were removed, HeLa cells washed x 2 to remove non-adherent BCBL-1 cells, and cultured an additional 24 h before harvest. Adherent HeLa cells were trypsinized, fixed and stained for intranuclear LANA dots. LANA^+^ HeLa cells were analyzed by MIFC. Graph shows percentage (mean ± s.d.) of infected cells from triplicate cultures for HeLa cells co-cultured with either rapamycin-treated (gray) or DMSO-treated (black) BCBL-1. ** p<0.01.

While RTA is necessary and sufficient for initiating viral lytic replication, it is only the initial measure of the productive phase of infection. Clinically, the most important read-out is the production of virions. To assess this parameter, we treated BCBL-1 cells with rapamycin, induced with VPA, TPA, or CoCl_2_ and then measured viral titers five days later. As we have shown previously in KSHV-infected cells, the number of punctate aggregates of virally-encoded latency-associated nuclear antigen (LANA), or “LANA dots”, correspond to the number of KSHV episomes present in the nucleus[38]. Thus, following de novo infection of KSHV-susceptible cells, the resulting LANA dots reflect the titer in the inoculum. Briefly, following each treatment we harvested media pre-cleared of cellular debris, collected the virus by centrifugation, and then incubated serial dilutions of the resuspended viral pellet with HeLa cells in the presence of polybrene. We measured titers of infectious virus by staining the KSHV-exposed HeLa cells for LANA and analyzing cells using multispectral imaging flow cytometry (MIFC) to determine the number of punctate LANA dots per cell. As evident in Figure 5A, rapamycin treatment decreased the amount of virus produced from BCBL-1 cells treated with VPA, TPA, or CoCl_2_ by 9.3, 9.7, and 7.7 fold, respectively.

Since spontaneous lytic reactivation likely better recapitulates viral propagation in the clinical setting, we assessed whether rapamycin similarly diminished virus release from uninduced BCBL-1 cells. The rate of spontaneous lytic reactivation, a correlate of virion release, is low in PEL cells in culture. We have previously found that de novo infection of naïve cells by KSHV is more efficient when the virus is cell-associated rather than cell-free and, thus, a more sensitive means of detecting low levels of infectious virion production in the laboratory setting [24]. Therefore, we assessed whether rapamycin was able to significantly decrease cell-to-cell transfer of KSHV from BCBL-1 to co-cultured HeLa cells. Mirroring our observations with the induced populations, we found that rapamycin also markedly decreased the levels of infectious virion production in spontaneously lytic BCBL-1 cells compared to titers from vehicle treated controls (Fig. 5C).

## Discussion

In this study we have shown that lytic reactivation of KSHV was partially controlled via mTOR signaling. Using a KSHV^+^ PEL line resistant to rapamycin-mediated growth inhibition, we demonstrated that in the absence of cell cycle arrest and death rapamycin was able to repress expression of the KSHV lytic master switch protein, RTA, and significantly impair virion production. Rapamycin, upon binding with FK506-binding protein 12 (FKBP12), inhibits several downstream functions of mTOR and, in susceptible cell types, can down-regulate mRNA translation and inhibit cell cycle causing G1 arrest [39]. More specifically, rapamycin can cause growth arrest in B cell lymphomas including subsets of EBV^+^ and of KSHV^+^ tumors [14], [40]–[42]. However, in contrast to many EBV infected [42] and uninfected B cells [40], [41], we found that in BCBL-1 cells rapamycin inhibition of mTOR function (reflected by the loss of S6K phosphorylation at Thr^389^) could be dissociated from significant arrest of cell cycle (Fig. 1) and loss of viability (Fig. 5). This finding is also in agreement with the work by Sin et al. that demonstrated that the responsiveness of KSHV-infected PEL cell lines to rapamycin growth arrest is highly variable [14]. By taking advantage of this cell line in which cell cycle arrest was dissociated from other rapamycin-mediated effects, we demonstrated that rapamycin treatment of cells inhibited the viral lytic cycle.

A wide range of DNA viruses activate the mTOR signaling pathway [43]. Herpesviruses, including KSHV, are well represented among this group. A primary pathway activating mTOR signaling involves PI3K-dependent growth factor signaling and both EBV and KSHV up-regulate the PI3K/Akt signaling pathway leading to mTOR activation [21], [44]–[47]. Activation of mTOR is also evident in KSHV-associated tumors [14], [15]. However, mTOR can receive information from a wide variety of upstream signaling pathways, including cytokine, growth factor, nutrient and stress sensors[48]. Work with EBV[42], [49] and the Sin et al. study [14] suggest that rapamycin might suppress KSHV-associated disease through non-virally associated cytokine dysregulation and control of tumor growth. The mechanism underlying the cellular resistance to rapamycin-mediated growth arrest, at least in a subset of PEL cell lines, remains unknown, though it might reflect the single or combined effects of several viral proteins expressed in infected cells that have the potential to dysregulate the cell cycle and disrupt apoptotic pathways [45], [50]–[56].

We have shown here that mTOR signaling is necessary for full expression of KSHV lytic proteins. Of interest, it has been previously demonstrated that vGPCR, also a lytic gene downstream of RTA, can upregulate mTOR activation, contributing to tumorigenesis via its promotion of growth and angiogenesis factors ([21], [57] and reviewed in [58]). Bottero et al. have more recently demonstrated that vGPCR can also enhance RTA transcription. Thus, the expression of vGPCR following the initiation of the lytic cycle likely enhances RTA levels by activating mTOR and enhancing SP1/SP3 mediated transcription of ORF50, providing a positive lytic feedback loop that may promote virion production [59]. It is conceivable that growth inhibition in rapamycin sensitive KSHV-infected tumors combined with the lytic cycle suppression that we have described in the rapamycin-resistant BCBL-1 line could act synergistically to result in an overall loss of productive infection and subsequent pathogenesis. The flip side of this synergism is that treatment with an mTOR inhibitor impacts KSHV tumors both by inhibiting tumorigenic growth, and angiogenesis, as well as by reducing virus production.

Our report is the first to show rapamycin sensitivity of viral replication for a herpesvirus, though a recent study has described the opposite finding: a rapamycin-insensitive mTOR-dependent replication pathway for HCMV [60]. These diverse results highlight the broad role that mTOR has as a downstream effector of many signaling pathways. We have examined the effects of rapamycin treatment on virus production and have shown that expression of KSHV RTA was rapamycin sensitive. Further, this repression of RTA expression, and ultimately virion production, occurred in a dose dependent manner irrespective of the induction pathway leading to the lytic cycle (Fig 2). Remarkably, rapamycin treatment also decreased the baseline levels of RTA expressed from the small percentage of cells undergoing spontaneous lytic reactivation (Figure 2A). These data suggested that mTOR plays an essential physiologic role in viral reactivation rather than being restricted to induction pathways initiated by the addition of exogenous chemical agents.

The exact mechanism(s) through which mTOR inhibition mediated its repressive effects on RTA expression was less clear. Our results suggest that the rapamycin-dependent inhibition of RTA might have occurred, in large part, at the level of translation, consistent with the role of rapamycin as a translation inhibitor [20]. We found that rapamycin dramatically decreased protein levels of RTA without requiring either concomitant global shutdown of cellular translation (Fig. 3A) or a decrease in ORF50 mRNA (Fig. 4). These findings were most evident when we tested the effects of rapamycin on cells induced with VPA. However, with either TPA- or CoCl_2_- induced cells, we also noted rapamycin-associated down-regulation of ORF50 mRNA. Previous work with HIV-1 and HTLV-1 has revealed that rapamycin-mediated inhibition of viral transcription results in decreased retroviral replication [61], [62]. For HTLV-1 this transcriptional regulation is linked to down-regulation of the transcription factor, c-Myb [61], rather than being a direct property of the mRNA itself as evident in the mTOR-mediated translational control of 5′-terminal oligo-pyrimidine-containing mRNA [63], [64]. Given the heterogeneity of the effects on ORF50 mRNA levels downstream of the different induction signals, it is likely that rapamycin treatment negatively affected one or more transcription factors. Thus, it is not surprising that rapamycin could have varied transcriptional effects in addition to post-transcriptional effects under the different induction conditions we examined.

Of note, recent work from Arias et al. demonstrated little effect of rapamycin on RTA expression in the TREx-RTA BCBL-1 line that are stably transduced with a doxycycline-inducible RTA construct. In this cell line, inducible RTA is robustly expressed from the CMV promoter [65], which has been shown to be rapamycin-insensitive in several systems[66], [67]. Low 4EBP1 phosphorylation reported in the TREx-RTA BCBL1 suggests that mTOR activity is also low in the line used for these studies, and may not reflect the mTOR activity of the parental line[68]. The group's finding that RTA could be post-transcriptionally down-regulated via blockade of Mnk activity is of particular importance, however, as it suggests an additional path with which to block lytic KSHV, particularly in patient tumors that are resistant to rapamycin inhibition.

Together, our results have provided a virally based explanation for the somewhat paradoxical clinical observations linking the immunosuppressant, rapamycin, to the control of the opportunistic malignancy, KS. Consistent with our findings, Barozzi et al. reported that the switch to a rapamycin-containing immunosuppressant regimen in a post-renal transplant patient led to a decrease in peripheral viral load that preceded any increase in T cell responses or the subsequent regression of KS lesions[22]. While anecdotal, this observation supports our data showing that rapamycin can suppress virus production. While further studies are necessary to elucidate the molecular details governing mTOR's role in RTA expression, our results have provided the first demonstration that rapamycin inhibited RTA expression and viral production. These findings have provided a potential molecular explanation for the anti-KS effects of rapamycin that further justify its continued study and use in preventing and treating KSHV-associated tumors in transplant patients.
